# Elevated sST2 associates with cardiac involvement and declines after treatment in newly diagnosed patients with idiopathic inflammatory myopathies

**DOI:** 10.1186/s13075-026-03830-w

**Published:** 2026-05-19

**Authors:** Balsam Hanna, Ingrid E. Lundberg, Anna-Karin H. Ekwall, Daniel Glinatsi, Egidija Sakiniene, Zhicheng Hu, Rille Pullerits, Tao Jin

**Affiliations:** 1 Department of Rheumatology and Inflammation Research, Institute of Medicine, Sahlgrenska Academy, University of Gothenburg, Gothenburg, Sweden; 2 Department of Rheumatology, Sahlgrenska University Hospital, Gothenburg, Region Västra Götaland Sweden; 3 Department of Medicine, Division of Rheumatology, Karolinska Institutet, Stockholm, Sweden; 4 Department of Gastroenterology, Dermatology and Rheumatology, Theme Inflammation and Aging, Karolinska University Hospital, Stockholm, Sweden; 5 Department of Rheumatology, Skaraborg Hospital, Skövde, Region Västra Götaland Sweden; 6 Center for Clinical Laboratories, Guizhou Medical University, Guiyang, China; 7 Department of Clinical Immunology and Transfusion Medicine, Sahlgrenska University Hospital, Region VästraGötaland, Gothenburg, Sweden

**Keywords:** Idiopathic inflammatory myopathies, Cardiovascular diseases, Magnetic resonance imaging, Biomarkers

## Abstract

**Objective:**

Cardiac involvement (CI) is a major determinant of poor prognosis in idiopathic inflammatory myopathies (IIM). Soluble suppression of tumorigenicity 2 (sST2) is a biomarker linked to cardiac inflammation and remodelling, but its role has scarcely been explored in IIM. We aimed to investigate the levels of sST2 and its longitudinal profile in patients with IIM and CI.

**Methods:**

Serum sST2 levels were measured longitudinally over the course of two years in 34 patients with newly diagnosed IIM and in 109 patients with established IIM from a cross-sectional cohort, as well as in age and gender matched healthy controls (HC) and in patients with rheumatoid arthritis (RA). CI was assessed using cardiac magnetic resonance (CMR) in newly diagnosed IIM patients. Associations between sST2 and clinical parameters were analyzed.

**Results:**

Patients with newly diagnosed IIM exhibited significantly higher sST2 levels at diagnosis compared to HC (*P* < 0.0001) and patients with established IIM (*P* < 0.0001). sST2 levels were highest at diagnosis, decreased significantly by 6 months, and remained lower during follow-up. sST2 levels were positively associated with cardiac troponin I and N-terminal pro-brain natriuretic peptide (*P* = 0.0001 and *P* = 0.0001, respectively). Importantly, IIM patients with active CMR-verified CI exhibited significantly higher sST2 levels than those without ongoing CI. In contrast, patients with established IIM had sST2 levels comparable to HC and RA patients.

**Conclusion:**

sST2 is elevated in early IIM and closely associated with cardiac biomarkers and CMR-verified cardiac inflammation. Its dynamic decline with treatment suggests potential utility as a biomarker for early detection and monitoring of CI in IIM.

**Supplementary Information:**

The online version contains supplementary material available at 10.1186/s13075-026-03830-w.

## Introduction

Idiopathic inflammatory myopathies (IIM) or shortly myositis, are chronic autoimmune disorders characterized by inflammation affecting skeletal muscle and, in many cases, extra-muscular organs, including the heart [1, 2]. Cardiac involvement in IIM may present as myocarditis, pericarditis or combined perimyocarditis [3]. Importantly, such involvement can be subclinical and therefore may remain underdiagnosed [4, 5]. Recent data suggest that cardiac involvement is associated with skeletal muscle involvement in newly diagnosed IIM. Although the long-term outcomes of cardiac involvement in IIM remain insufficiently understood, several studies indicate an association with later development of heart failure. Indeed, heart failure is frequently reported in IIM patients [3], and those with polymyositis (PM) or dermatomyositis (DM) have a more than two-fold increased risk of developing heart failure compared with population controls (HR 2.06) [6]. Furthermore, cardiac involvement has been identified as a significant prognostic factor for mortality in a long-term follow-up study [7].

Soluble suppression of tumorigenicity (sST2) is a biomarker elevated in several cardiac conditions, including heart failure and myocardial infarction [8, 9]. Elevated blood levels of sST2 impair adaptive cardiac remodeling signals [10, 11], contribute to development of heart failure [12], and are associated with poor outcome [13]. Released in response to vascular congestion, inflammatory and pro-fibrotic stimuli, sST2 is a strong predictor of mortality and hospitalization in both acute and chronic heart failure [14]. sST2 belongs to the interleukin-1 family and is part of a ST2/IL-33 pathway, which comprises IL-33 and two different isoforms of suppression of tumorigenicity 2 receptors: soluble form (sST2) and transmembrane form (ST2L) [15–17]. Binding between ST2L and IL-33 promotes a cardioprotective mechanism, whereas sST2 acts as a decoy receptor, neutralizing IL-33 and thereby silencing this protective signaling [16, 18, 19]. Interestingly, a recent cross-sectional study has shown that elevated sST2 levels are associated with diffuse and focal myocardial fibrosis verified by cardiac magnetic resonance (CMR) assessment in patients with IIM [20].

Given the significant role of sST2 in cardiovascular diseases, we hypothesize that elevated sST2 may reflect cardiac involvement or overt cardiac damage in patients with IIM and could potentially serve as a biomarker for cardiac involvement in this population. However, data on sST2 in IIM, particularly in relation to cardiac manifestations, remain limited, and its potential as a biomarker for early detection has not been evaluated. The aim of the current study was to investigate the role of sST2 in IIM and its potential association with cardiac involvement in the disease.

## Materials and methods

### Patients and healthy controls

This is a single-center study, in which two IIM patient cohorts were included.

The first prospective cohort consists of newly diagnosed patients with IIM enrolled between February 2020 to September 2022 at the Rheumatology Clinic, Sahlgrenska University Hospital, Gothenburg, Sweden. The study was undertaken to study cardiac involvement in IIM. Newly diagnosed IIM patients undergoing CMR investigation and healthy controls (*n* = 20) matched with the patient group in terms of age, gender, and ethnicity were included. The patients were eligible for inclusion if they met the 2017 European Alliance of Associations for Rheumatology/American College of Rheumatology (EULAR/ACR) classification criteria for IIM [21] or fulfilled Connors' criteria for anti-synthetase syndrome (ASyS) [22] or criteria for overlap myositis [23] and had diagnosis and initiation of treatment for IIM less than two months prior to enrollment. Patients were classified using a scoring system according to 2017 EULAR/ACR criteria as follows: definite IIM (total score ≥ 7.5 without and ≥ 8.7 with muscle biopsy), probable IIM (≥ 5.5 without and ≥ 6.7 with biopsy), and possible IIM (≥ 5.3 without and ≥ 6.5 with biopsy). CMR was performed according to a standardized protocol enabling the visualization of cardiac inflammation in accordance with current recommendations [24]. Image analysis evaluated in a blinded manner by an experienced imaging expert. The presence of cardiac involvement, in form of myocarditis and/or pericarditis was based on the Updated Lake Louise Criteria [25]. Consequently, the presence of myocardial inflammation was based on the proposed “2 out of 2” approach, which means that one positive T2-based criterion (T2-weighted imaging or T2 mapping) and one T1-based criterion (T1 mapping, extracellular volume, or Late Gadolinium Enhancement, (LGE)) must be fulfilled. The detailed information about these 34 IIM patients and 20 healthy controls regarding inclusion and exclusion criteria, data collection, laboratory analysis and the results is previously reported [26]. These 34 newly diagnosed IIM patients were prospectively followed up at 6, 12 and 24 months.

To investigate disease specificity of sST2, blood samples from a cohort of patients with newly diagnosed rheumatoid arthritis, rheumatoid factor and/or anti-citrullinated protein antibody positive, active disease, who were matched with the prospective study group of IIM patients in terms of age and gender were included.

The second cross-sectional established IIM cohort consists of 109 IIM patients identified from patient registries during 2003–2022 with established IIM, diagnosed more than 6 months ago. To be included, the patients had to meet classification criteria for definite or probable PM/DM according to Bohan & Peter [27, 28], or fulfil Connors' criteria for ASyS [22] or criteria for overlap myositis [23]. For this cohort, the 2017 EULAR/ACR classification criteria for IIM were not adopted since many patients were diagnosed with IIM long before the EULAR/ACR criteria became available. The information about study design, inclusion and exclusion criteria, data collection, laboratory analysis and results were reported previously [29].

Serum samples from healthy individuals recruited as controls to the prospective IIM study (*n* = 20) and healthy blood donors (*n* = 69) were age- and sex-matched to the cross-sectional established IIM patient cohort (*n* = 109) and used as the healthy control reference group (*n* = 89).

### Data collection

All patients and healthy controls in the prospective and cross-sectional cohorts were evaluated and enrolled into the studies by the same physician (Balsam Hanna). Clinical and laboratory data were collected as previously described [26, 29].

Disease activity was assessed according to the Core Set Measures of the International Myositis Assessment & Clinical Studies Groups (IMACS) [30] using the physician's global assessment of disease activity (PGA), patient`s global assessment of disease activity, extra muscular disease activity score, the standardized manual muscle test 8 (MMT-8), the Health Assessment Questionnaire Disability Index (HAQ-DI) and serum level of muscle enzymes (creatine kinase (CK), lactate dehydrogenase, myoglobin, alanine aminotransferase, aspartate aminotransferase) [31]. Cardiac injury/function markers (as cardiac troponin I (cTnI) and N-terminal pro-brain natriuretic peptide (NT-proBNP)) were also collected.

The venous blood samples were collected at inclusion and at follow-up for routine clinical-laboratory analyses. The serum aliquots were stored at −80 C for further analysis of biomarkers.

All study participants completed a comprehensive questionnaire (including inquiries about cardiovascular comorbidities, ongoing medical treatments, patient's global assessment of disease activity and the standardized HAQ-DI. The study was conducted according to the Declaration of Helsinki and approved by the Swedish Ethical Review Authority (Dnr. 2019–04253) and all included patients and healthy controls signed a written consent for participation in the study.

### Detection of soluble ST2

The serum concentrations of sST2 were measured using Quantikine® ELISA human S2/IL-33R immunoassay (R&D Systems, Minneapolis, MM, USA). ELISA plates coated with a monoclonal antibody specific for human ST2 were used for quantification. The patient samples were diluted 1:30 or 1:100, as appropriate, in sample diluent. After incubation for 2 h at room temperature and wash, polyclonal detection antibody specific for human ST2 conjugated to horseradish peroxidase was used for incubation during next two hours. The plates were thereafter washed and incubated 30 min at dark with substrate solution consisting of tetramethylbenzidine and stabilized hydrogen peroxide. After adding hydrochloric acid to stop the reaction, the optical density was determined at 450 nm using wavelength correction at 570 nm. The mean minimum detectable dose of sST2 was 5,1 ng/ml, intra-assay precision (CV%) 4,4–5,6 and inter-assay precision (CV%) 5,4–7,1 according to manufacturer.

### Statistical analysis

Statistical analyses were performed using GraphPad Prism (version 10.0.0). Descriptive statistics for continuous variables are presented as the median with interquartile range (IQR), and for categorial variables as the number and/or the percentage. For continuous variables, the differences between the patient group and control group were assessed by using Student`s *t* test (normal distribution) or Mann–Whitney U test (non-normal distribution). For categorial data, the differences in frequencies were calculated by chi-square test. For comparison among three groups, statistical analyses were performed using Kruskal–Wallis test with Dunn's post-test. Within-group changes over time were analysed using paired t-tests for normally distributed variables and Wilcoxon signed-rank tests for non-parametric variables. A correlation between sST2 and other variables was examined by the Spearman`s correlation test or Pearson´s correlation test, as appropriate. A two tailed p value < 0.05 was considered statistically significant.

## Results

### Patient characteristics

A summary of demographic and disease-related characteristics of newly diagnosed IIM patients and patients with established IIM disease from cross-sectional cohort is presented in Table 1 (Table 1 located after references). The newly diagnosed IIM cohort (*n* = 34) comprised 53% females and had a median age of 67 years (IQR 52–72), while the established IIM cohort comprised 61% females and a median age of 60 years (IQR 45–70). As expected, newly diagnosed IIM exhibited more frequent clinical manifestations and higher disease activity.

**Table 1:** The demographic and disease-related characteristics of 34 patients with newly diagnosed idiopathic inflammatory myopathies (IIM) and patients with established IIM cohort (*n* = 109)

Characteristics Newly diagnosed IIM (*n* = 34)		*Established IIM (*n* = 109)
Age years, median (IQR)	67 (52–72)	60 (45–70)
Gender, female, n (%)	18 (53)	67 (61)
Ethnicity, n (%)		
Caucasian	32 (94)	105 (96)
Asian/African	2/0 (6)	2/2 (4)
Disease duration in months, median (IQR)	-	32 (12–52)
IIM subtype, n (%)		
IMNM	13 (38)	26 (24)
Anti-synthetase syndrome	10 (29)	38 (36)
Dermatomyositis	7 (21)	23 (20)
Overlap myositis	3 (9)	17 (15)
Polymyositis	1 (3)	5 (5)
Autoantibody profile, n (%)		
MSA	26 (76)	83 (76)
MAA	4 (12)	36 (33)
Seronegative	6 (18)	14 (13)
Clinical manifestation at the time of enrolment, n (%)		
Myositis	25 (74)	17 (16)
Interstitial lung disease	18 (53)	7 (6)
Skin manifestation	12 (35)	14 (13)
Dysphagia	14 (41)	6 (6)
Arthritis	10 (29)	3 (3)
Disease activity measures, median (IQR)		
Physician global disease activity VAS, 0–100	60 (47–60)	10 (10–13)
Patient global disease activity VAS, 0–100	80 (67–82)	25 (23–25)
Extramuscular disease activity score VAS, 0–100	50 (20–60)	0 (0–10)
HAQ-DI, 0–3	0.56 (0–1.28)	0 (0–0.63)
Manual muscle test, 0–80	75 (67–80)	80 (80–80)
CK, µkat/L, median (IQR)	16 (1.2–61.5)	1.5 (1.0–2.6)

*CK* Creatine kinase, *HAQ-DI* Health Assessment Questionnaire-disability index, *IQR* Interquartile range, *IIM* Idiopathic inflammatory myopathies, *IMNM* Immune-mediated necrotizing myopathies, *MSA* Myositis specific antibody, *MAA* Myositis associated antibody, *VAS* Visual analog scale

^*^Established IIM: IIM patients with diagnosis for more than 6 months

The information about cardiovascular risk factors and comorbidities in patients with newly diagnosed and established IIM is presented in Supplementary Table S1.

In the RA cohort, 56% were female, with a median age of 65 years (IQR 51–72) and a median disease activity score (DAS28) of 4.6 (IQR 4.7–5.7).

### High sST2 levels and change over time

**Fig. 1:**
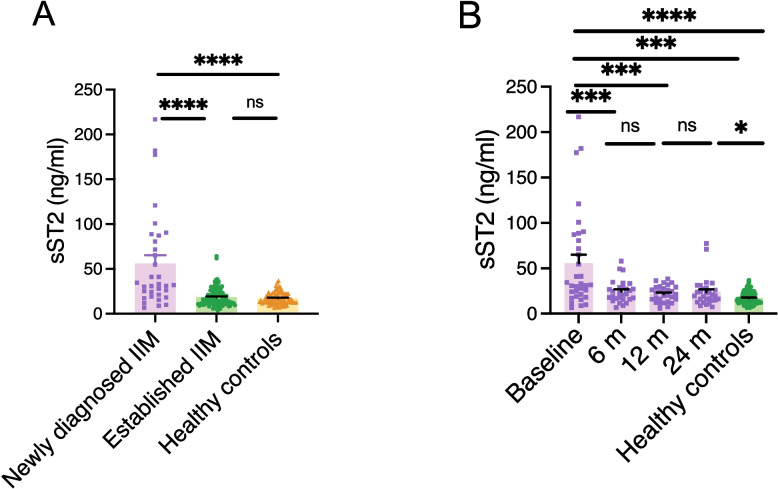
Serum sST2 levels in newly diagnosed and established IIM over time. **A** Serum levels of sST2 in patients with newly diagnosed IIM (*n* = 34) compared to IIM with established disease (*n* = 109) and healthy controls (*n* = 89). **B** Serum levels of sST2 in patients with newly diagnosed IIM (*n* = 34) at baseline and after 6, 12, and 24 months, as well as in comparison with healthy controls (*n* = 89). IIM: idiopathic inflammatory myopathies. * *P* < 0.05, ** *P* < 0.01, *** *P* < 0.001, **** *P* < 0.0001, ns = non-significant

Serum median sST2 levels were significantly higher in the 34 newly diagnosed IIM patients at inclusion (32, IQR 20–84 ng/ml) than in 109 patients with established IIM (14, IQR 11–21 ng/ml, *P* < 0.0001) and in the 89 healthy controls (16, IQR 12–22 ng/ml, *P* < 0.0001), respectively. Of note, the median sST2 concentration in patients with established IIM disease was almost identical to healthy controls (Fig. 1A). Given the substantial difference in disease activity between the newly diagnosed and established IIM cohorts, this may confound the observed differences in sST2 levels. We therefore performed a logistic regression analysis adjusting for PGA when comparing these two groups. After adjustment, the difference in sST2 levels was no longer statistically significant, suggesting that the elevated sST2 levels observed in newly diagnosed IIM are largely driven by overall disease activity. Consistent with this, sST2 levels were significantly correlated with PGA in the newly diagnosed cohort, whereas no such correlation was observed in the established IIM cohort, despite its larger sample size.

In patients with newly diagnosed IIM, the median sST2 levels were significantly higher (*P* < 0.0001) at baseline compared to the corresponding sST2 levels at 6 months (22, IQR 16–29), 12 months (21, IQR 14–29) and 24 months (20, IQR 15–28) (Fig. 1B). In parallel, a significant decline in disease activity was observed over time, accompanied by changes in treatment patterns across the follow-up periods (Supplementary Table S2).

### Newly diagnosed IIM patients with ongoing Myocarditis and/or pericarditis exhibited significantly higher sST2 levels compared to patients without ongoing myocarditis/perimyocarditis

**Fig. 2:**
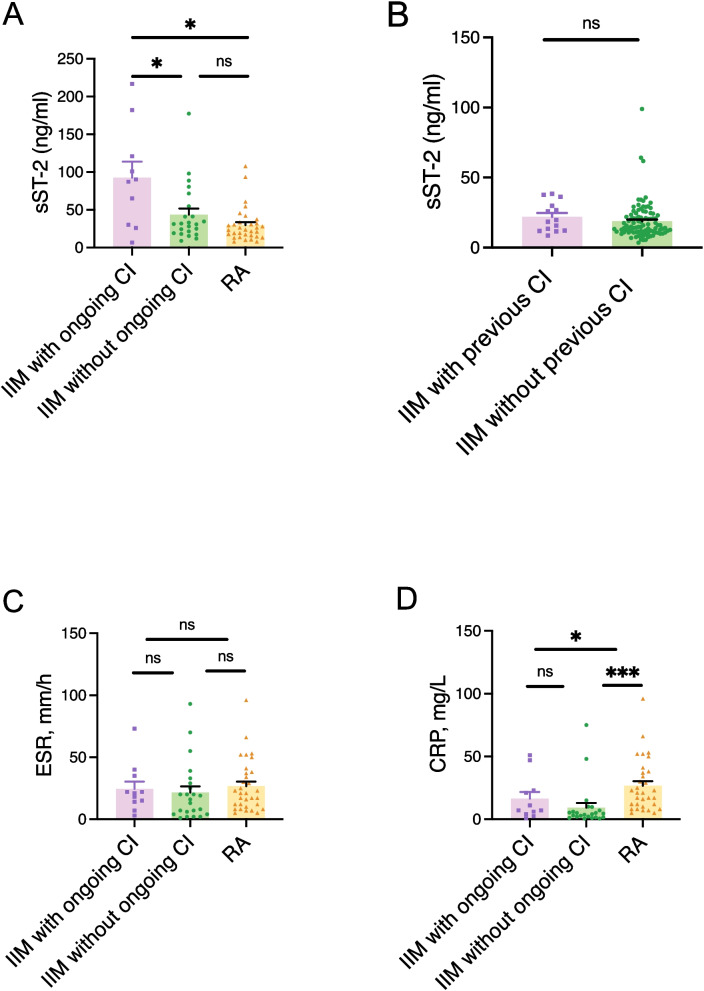
Baseline sST2 levels and inflammatory markers in IIM with and without CI versus RA. **A** Baseline sST2 levels in patients with newly diagnosed IIM and ongoing CI (*n* = 11) were significantly higher compared with those in patients with newly diagnosed IIM without ongoing CI (*n* = 23) and in patients with newly diagnosed RA (*n* = 32). **B** Among patients with established IIM, sST2 levels did not differ between those with (*n* = 14) and without (*n* = 95) previous clinical CI. **C** ESR levels did not differ among patients with newly diagnosed IIM with ongoing CI (*n* = 11), without ongoing CI (*n* = 23), and those with RA (*n* = 32). **D** CRP levels were significantly higher in patients with newly diagnosed RA (*n* = 32) compared with IIM patients with (*n* = 11)and without (*n* = 23) CI. CI: cardiac involvement, defined as the presence of myocarditis and/or pericarditis by cardiac magnetic resonance; CRP: C-reactive protein; ESR: erythrocyte sedimentation rate; IIM: idiopathic inflammatory myopathies; RA: rheumatoid arthritis. * *P* < 0.05, *** *P* < 0.001, ns = non-significant

Out of 34 newly diagnosed IIM, 11 patients showed changes in CMR, indicating ongoing myocarditis and/or pericarditis, and these patients exhibited significantly higher sST2 levels (P = 0.022, median 89, IQR 29–136) compared to newly diagnosed IIM without ongoing myocarditis and/or pericarditis (n = 23), Fig. 2A. Of note, none of the patients with ongoing myocarditis and/or pericarditis had a prior history of ischemic heart disease or heart failure. Furthermore, patients with newly diagnosed rheumatoid arthritis had similar levels of sST2 (24, IQR 15–33 ng/ml) as newly diagnosed IIM patients without ongoing cardiac involvement (31, IQR 18–41 ng/ml) despite having higher systemic inflammation as shown by significantly elevated C-reactive protein (CRP) (Fig. 2D). Regarding established IIM cohort, there were no significant differences between IIM patients with previous clinical cardiac involvement and IIM patients without previous clinical cardiac involvement (P = 0.128). (Fig. 2B).

These results indicate that elevated sST2 levels are not merely a reflection of inflammatory activity but may be more specific to active cardiac involvement.

### sST2 levels were positively associated with cardiac biomarkers at disease onset and related to long-term comorbidities

Next, we investigated sST2 levels and association with cardiac involvement, cardiac biomarkers, and overall disease activity. In patients with newly diagnosed IIM (at baseline), the levels of sST2 showed a statistically significant positive correlation with PGA and extra muscular disease activity as expressed by VAS. Of importance, at the time of IIM diagnosis a significant strong positive correlation was observed between sST2 levels and serum levels of cardiac biomarkers: cTnI (*P* < 0.0001, rho 0.652) and NT-proBNP (*P* < 0.0001, rho 0.740). Furthermore, the sST2 levels were significantly positively associated with CMR-verified signs of ongoing myocarditis and/or pericarditis (*P* = 0.001, rho 0,536) (Table 2). In IIM patient samples with established IIM disease, a significant, but weaker correlation was observed between sST2 levels and cardiac biomarkers.

**Table 2:** Correlations between sST2 levels and different disease variables in newly diagnosed IIM (*n* = 34) and in IIM patients with established disease from the cross-sectional cohort (*n* = 109)

Variables	Newly diagnosed IIM, *n* = 34 P-value (r)	*Established IIM, *n* = 109 P-value (r)
Age, years	0.646 (0.207)	**0.003 (0.278)**
Sex, male	0.206 (0.226)	**0.001 (0.305)**
BMI, kg/m^2^	0.439 (−0.139)	0.193 (0.125)
Diabetes mellitus	0.206 (0.225)	**0.003 (0.276)**
Hypertension	0.120 (0.275)	**0.009 (0.249)**
Dyslipidaemia	0.266 (0.199)	**0.0005 (0.329)**
Myositis	0.102 (0.289)	**0.0005 (0.329)**
Interstitial lung disease	0.470 (0.130)	0.492 (0.665)
Arthritis	0.158 (−0.251)	**0.023 (0.216)**
Dysphagia	0.803 (−0.045)	0.788 (−0.162)
Skin involvement	0.208 (−0.224)	0.059 (0.181)
PGA VAS, 0–100	**0.010 (0.441)**	0.070 (0.174)
PAGA VAS, 0–100	0.979 (0.004)	**0.017 (0.227)**
Extramuscular disease activity VAS, 0–100	**0.015 (0.417)**	0.581 (0.053)
HAQ-DI, 0–3	0.154 (0.253)	0.191 (0.126)
MMT-8, 0–80	0.124 (−0.272)	0.472 (−0.069)
CK, ukat/L	0.231 (0.214)	0.136 (0.143)
Myoglobin, µg/L	0.082 (0.308)	**0.030 (0.207)**
cTnI, ng/L	** < 0.0001 (0.652)**	**0.005 (0.263)**
NT-proBNP, ng/L	** < 0.0001 (0.740)**	**0.006 (0.258)**
Sign of ongoing myocarditis and/or pericarditis by CMR	**0.001 (0.536)**	**-**

*Bold values indicate p<0.05*

*AST* Aspartate aminotransferase, *CK* Creatine kinase, *CMR* Cardiac magnetic resonance, *cTnI* cardiac troponin I, *IIM* Idiopathic inflammatory myopathies, *NT-proBNP* N-terminal pro-brain natriuretic peptide, *PGA* Physician global assessment of disease activity, *PAGA* Patient global assessment of disease activity, *r* spearman`s Rho or Pearson´s as appropriate

^*^Established IIM were IIM patients with diagnosis more than 6 months

In the newly diagnosed IIM patients, the baseline sST2 levels did not correlate with age, sex, presence of cardiovascular comorbidities (diabetes mellitus, hypertension and dyslipidemia) or clinical manifestations of IIM. In contrast, in IIM patients with established disease, sST2 levels correlated significantly with age, male sex, cardiovascular comorbidities, and clinical manifestations such as myositis and arthritis (Table 2).

### cTnI and NT-ProBNP levels were significantly higher in IIM patients with the highest sST2 levels at early stage of the disease

**Fig. 3:**
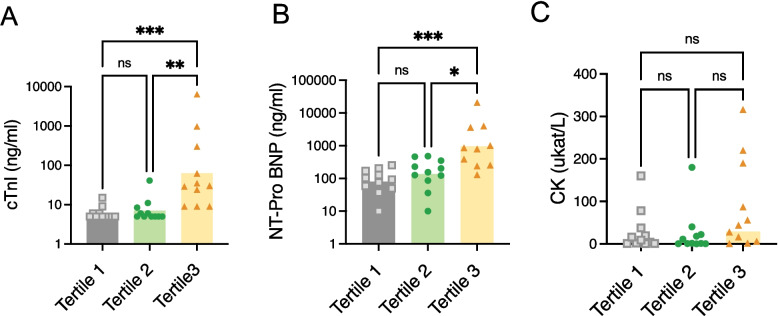
Association of sST2 tertiles with cardiac and muscle involvement biomarkers in newly diagnosed IIM. **A** cTnI levels in patients with newly diagnosed IIM were significantly higher in the high sST2 tertile group compared with those in the low and middle tertile groups. **B** NT-proBNP levels in patients with newly diagnosed IIM were significantly higher in the high sST2 tertile group compared with those in the low and middle tertile groups. **C** CK levels did not differ among the tertile groups. Tertile 1 represents the lowest sST2 levels, tertile 2 represents intermediate levels, and tertile 3 represents the highest sST2 levels. CK: creatine kinase; cTnI: cardiac troponin I; IIM: idiopathic inflammatory myopathies; NT-proBNP: N-terminal pro–B-type natriuretic peptide. * *P* < 0.05, ** *P* < 0.01, *** *P* < 0.001, ns = non-significant

We thereafter investigated more closely what characterizes the IIM patients displaying the highest levels of sST2 at diagnosis. The newly diagnosed IIM patients were tertile-stratified into three groups according to sST2 values and thereafter cTnI, NT-proBNP and disease activity measures were compared between the tertile groups. Interestingly, newly diagnosed IIM patients belonging to the high sST2 tertile group (Tertile 3) exhibited a significantly higher cTnI and NT-proBNP levels compared to patients in the low (*P* = 0.0004 and *P* = 0.0004, respectively) and middle tertile groups (*P* = 0.001 and *P* = 0.020, respectively) (Fig. 3). There were no significant differences between tertile groups regarding CK levels. Although limited by the small sample size and therefore considered exploratory, these findings further support an association between elevated sST2 and active cardiac involvement.

Regarding disease activity measures, the highest sST2 tertile group (Tertile 3) exhibited a significantly higher PGA compared to the lowest sST2 tertile group (tertile 1, *P* = 0.01) but the other disease activity measures did not differ between the tertile groups. (Supplementary Figure S1).

### Sex-based differences in sST2 levels

Studies have indicated that sex-based differences exist in sST2 levels and healthy men usually have higher sST2 levels as compared to corresponding levels in women [32]. In the newly diagnosed IIM patients, there were no significant differences in sST2 levels between male and female patients (41, IQR 25–90 ng/ml and 29, IQR 17–71 ng/ml, respectively, P = 0.155). This finding remained unchanged after adjustment for disease activity (PGA). However, male patients with established IIM had significantly higher sST2 levels as compared to females (20, IQR 13–27 ng/ml and 14, IQR 10–21 ng/ml, respectively, p = 0.001).The sex differences were also significant in healthy controls (men 23, IQR 15–26 ng/ml and women 18, IQR 13–31 ng/ml, p < 0.0001) (Supplementary Figure S2).

## Discussion

In this study, we could demonstrate that serum sST2 levels were significantly elevated in newly diagnosed IIM patients compared to healthy controls, and in particular in patients with clinically evident cardiac involvement and correlated primarily with cardiac biomarkers. Importantly, sST2 levels had declined markedly within six months of treatment initiation and eventually stabilized, suggesting its value in monitoring treatment response. In contrast, sST2 levels in our established IIM cohort of patients with stable, well-controlled disease were comparable to those of healthy individuals. Notably, sST2 levels showed consistent correlations with cardiac biomarkers, cTnI and NT-proBNP, in both newly diagnosed and established IIM patients’ groups. While in the established group, sST2 was also associated with age and traditional cardiovascular risk factors, including diabetes, hypertension, and dyslipidaemia. The higher serum levels of sST2 in newly diagnosed IIM patients with evident cardiac involvement on CMR, further support the potential of sST2 as a biomarker for cardiac inflammation in IIM.

Elevated sST2 levels have previously been reported in several rheumatic diseases, including rheumatoid arthritis, systemic lupus erythematosus (SLE), and Sjögren’s disease in relation to inflammatory active disease. In rheumatoid arthritis, sST2 levels were higher than in osteoarthritis patients [33]. In SLE and Sjögren’s disease, sST2 levels were associated with disease activity and have been proposed as a potential marker for lupus nephritis [34–38]. In line with these findings, our results show that sST2 levels are elevated in newly diagnosed inflammatory active IIM and decline with treatment, reinforcing its potential role as a marker of disease activity and therapeutic response. In our newly diagnosed IIM cohort, sST2 levels correlated with PGA, extramuscular disease activity score, and cardiac biomarkers, as well as with CMR findings indicative of active myocardial inflammation. However, sST2 did not correlate with classical skeletal muscle inflammation markers such as CK or myoglobin, suggesting that sST2 may not reflect muscle inflammation directly. Furthermore, in our pervious study [26] (same newly diagnosed IIM cohort), cTnI levels were also correlated with ongoing myocardial inflammation in IIM patients with active muscle involvement. As cTnI is specific for myocardial involvement, this further supports the hypothesis that elevated sST2 levels are more indicative of cardiac involvement than of overall disease activity.

The IL-33/ST2 signalling axis has emerged as a key modulator in cardiovascular pathophysiology. IL-33, produced mainly by cardiac fibroblasts under mechanical stress, exerts protective effects by antagonizing angiotensin II- and phenylephrine-induced cardiomyocyte hypertrophy [19, 39, 40]. These effects are counteracted by sST2, which acts as a decoy receptor. In murine models of atherosclerosis, IL-33 administration reduces plaque burden, whereas sST2 promotes disease progression [41]. In IIM, sST2 levels have been shown to correlate with both focal and diffuse myocardial fibrosis, suggesting a role in the early detection of fibrotic cardiac involvement [20]. Additionally, myocardial inflammation can independently drive sST2 elevation. In murine models of myocarditis, sST2 levels correlated with cardiac IL-1β expression and overall inflammation [42]. Furthermore, in clinical settings, sST2 distinguishes fulminant from non-fulminant myocarditis and unrelated acute heart failure, and longitudinal monitoring reveals that sST2 levels decline with resolution of inflammation [43]. In our study, three newly diagnosed IIM patients with overt cardiac involvement exhibited sST2 levels nearly ten times higher than the mean levels in those without cardiac inflammation, highlighting its potential diagnostic value in identifying severe cardiac manifestations in IIM but this observation needs to be validated in a larger cohort. At the same time, our findings do not establish whether elevated sST2 in IIM reflects cardiac inflammation, myocardial stress, or broader systemic inflammation. Further mechanistic studies are warranted to clarify the biological basis of sST2 elevation in IIM.

sST2 levels are also influenced by traditional cardiovascular risk factors. In genetically obese diabetic mice, IL-33 treatment reduced adiposity, improved insulin sensitivity, and decreased fasting glucose, whereas ST2-deficient mice developed greater adiposity and metabolic dysfunction [44]. Elevated sST2 concentrations have also been reported in humans with metabolic syndrome, left ventricular hypertrophy [45], diabetes [46, 47], and hypertension [48]. Consistent with these findings, we observed significant associations between sST2 and traditional cardiovascular risk factors in our established IIM cohort.

Interestingly, only newly diagnosed IIM patients exhibited significantly elevated sST2 levels compared to healthy controls, whereas patients with established IIM did not. This finding contrasts with prior studies reporting elevated sST2 levels in cross-sectional IIM cohorts [49, 50]. The discrepancy is unlikely to be due to differences in sample size, as our study included a slightly larger cohort. Instead, variation in disease duration likely explains the difference: our established IIM patients had a median disease duration of 32 months. Supporting this, longitudinal data from our newly diagnosed cohort showed persistently elevated sST2 levels up to two years post-diagnosis, particularly in those with cardiac involvement.

The distinct correlation patterns observed in the two cohorts further highlight the context-dependent nature of sST2 elevation. In newly diagnosed IIM, sST2 levels correlated strongly with cardiac biomarkers, while in established IIM disease, associations extended to metabolic factors. This suggests that during the acute inflammatory phase, cardiac involvement is the dominant driver of sST2 elevation, which may mask contributions from other factors. A similar pattern was observed with sex, although men typically have higher sST2 levels [42], this sex difference was only observed in the established IIM cohort and not among newly diagnosed patients, likely due to the overwhelming influence of acute cardiac inflammation. Additionally, despite that patient with newly diagnosed rheumatoid arthritis showed higher systemic inflammation as indicated by significantly elevated CRP levels, patients with newly diagnosed IIM and ongoing cardiac involvement exhibited significantly higher sST2 levels compared with patients with newly diagnosed rheumatoid arthritis.

The current study has limitations. First, the newly diagnosed cohort was relatively small (n = 34), with only 11 patients having cardiac inflammation confirmed by CMR, limiting power for some subgroup analyses. However, the inclusion of two-year longitudinal data strengthens the conclusions. Second, the cross-sectional cohort with established IIM disease was classified using older Bohan and Peter criteria, while for the newly diagnosed IIM cohort we used updated EULAR/ACR 2017 criteria. The difference in classification criteria might introduce systematic variations in clinical phenotype, disease severity, and treatment background, and may potentially influence direct comparison of sST2 levels between newly diagnosed and established IIM that should be interpreted with caution. However, the observed between-cohort difference disappeared after adjustment for PGA, supporting the interpretation that active disease rather than classification criteria contributed to the higher sST2 levels at diagnosis. Third, systematic CMR assessment was only available in the newly diagnosed cohort, whereas cardiac involvement in the established cohort was assessed based on clinical evaluation. Therefore, subclinical cardiac involvement may have been underdetected in the established cohort, which may affect the comparison of sST2 levels in relation to cardiac involvement between the two groups. Finally, systematic follow-up CMR data were not available, making it impossible to assess whether the decline in sST2 paralleled improvement in CMR abnormalities.

In conclusion, our findings suggest sST2 as a potential biomarker for cardiac involvement in IIM, in the early phase of disease. Its associations with cardiac biomarkers and traditional CV risk factors suggest that sST2 may also serve as a marker of subclinical cardiac stress and long-term cardiovascular risk. Future large-scale, longitudinal studies are warranted to assess the prognostic value of sST2 and explore whether sST2-guided monitoring could improve early detection and management of cardiac complications in IIM.

## Supplementary Information

Supplementary Material 1. Supplementary Figure S1. Association of sST2 tertiles with disease activity measures in newly diagnosed idiopathic inflammatory myopathies.
(A) PGA in patients with newly diagnosed IIM were significantly higher in the high sST2 tertile group (tertile 3) compared with those in tertile 1. However, neither PAGA (B), extramuscular disease activity (C), HAQ-DI (D), nor MMT-8 (E) differed among the tertile groups. PGA: physician global assessment of disease activity; PAGA: patient global assessment of disease activity; HAQ-DI: Health Assessment Questionnaire–Disability Index; MMT-8: manual muscle testing–8. * *P* < 0.05.

Supplementary Material 2. Supplementary Figure S2. Differences in sST2 levels between male and female participants.
(A) newly diagnosed IIM (*n* = 34), (B) IIM with established IIM disease (*n* = 109), and (C) healthy controls (*n* = 89). IIM: idiopathic inflammatory myopathies. ** *P* < 0.01, **** *P* < 0.0001, ns = non-significant.

Supplementary Material 3. Supplementary Table S1. Cardiovascular comorbidities and risk factors in patients with newly diagnosed IIM and patients with established IIM.

Supplementary Material 4. Supplementary Table S2. Disease activity and treatment for 34 newly diagnosed IIM patients at baseline, 6 months,12 months and 24 months follow-up.

## Data Availability

The datasets used and/or analyzed during the current study are available from the corresponding author on reasonable request.

## Abbreviations

ASyS: Anti-synthetase syndrome
ACR: American College of Rheumatology
CK: Creatine kinase
CMR: Cardiac magnetic resonance
CRP: C-reactive Protein
DM: Dermatomyositis
EULAR: European Alliance of Associations for Rheumatology
cTnI: Cardiac troponin I
HAQ-DI: Health Assessment Questionnaire Disability Index
IIM: Idiopathic inflammatory myopathies
IQR: Interquartile range
IMACS: International Myositis Assessment & Clinical Studies Groups
LGE: Late Gadolinium Enhancement
MMT-8: Manual muscle test 8
PM: Polymyositis
PGA: Physician global assessment of disease activity
NT-proBNP: N-terminal pro-brain natriuretic peptide
SLE: Systemic lupus erythematosus
sST2: Soluble suppression of tumorigenicity
