# Supplementary figures and images for: Elevated sST2 associates with cardiac involvement and declines after treatment in newly diagnosed patients with idiopathic inflammatory myopathies
Source: Arthritis Res Ther. 2026 May 19;28:109. doi: 10.1186/s13075-026-03830-w (PMC13185292; doi:10.1186/s13075-026-03830-w)

A

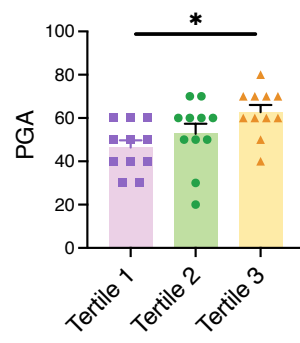

B

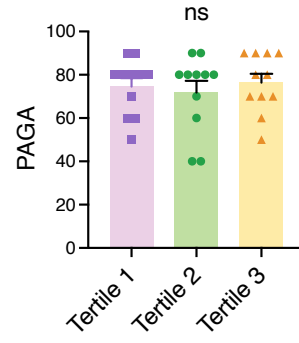

C

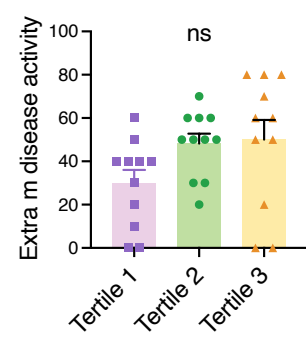

D

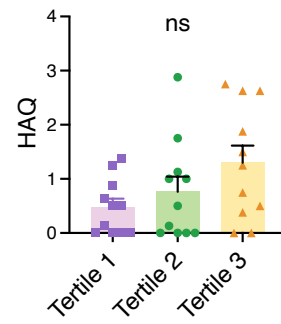

E

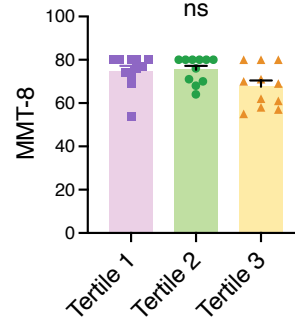

Supplement: Supplementary file 1 — Supplementary Material 1. Supplementary Figure S1. Association of sST2 tertiles with disease activity measures in newly diagnosed idiopathic inflammatory myopathies. (A) PGA in patients with newly diagnosed IIM were significantly higher in the high sST2 tertile group (tertile 3) compared with those in tertile 1. However, neither PAGA (B), extramuscular disease activity (C), HAQ-DI (D), nor MMT-8 (E) differed among the tertile groups. PGA: physician global assessment of disease activity; PAGA: patient global assessment of disease activity; HAQ-DI: Health Assessment Questionnaire–Disability Index; MMT-8: manual muscle testing–8. * P < 0.05. [file 13075_2026_3830_MOESM1_ESM.pdf]

A

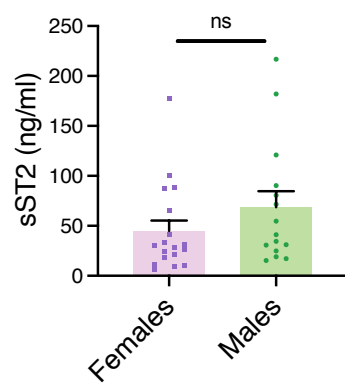

B

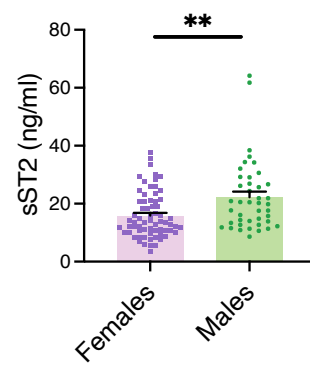

C

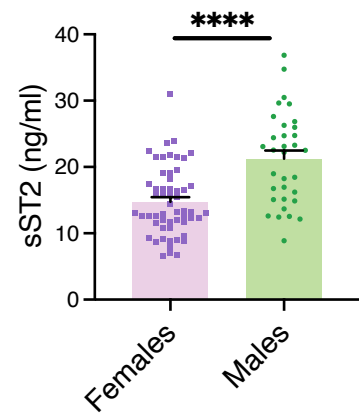

Supplement: Supplementary file 2 — Supplementary Material 2. Supplementary Figure S2. Differences in sST2 levels between male and female participants. (A) newly diagnosed IIM (n = 34), (B) IIM with established IIM disease (n = 109), and (C) healthy controls (n = 89). IIM: idiopathic inflammatory myopathies. ** P < 0.01, **** P < 0.0001, ns = non-significant. [file 13075_2026_3830_MOESM2_ESM.pdf]
