# Supplementary material for: Elevated sST2 associates with cardiac involvement and declines after treatment in newly diagnosed patients with idiopathic inflammatory myopathies
Source: Arthritis Res Ther. 2026 May 19;28:109. doi: 10.1186/s13075-026-03830-w (PMC13185292; doi:10.1186/s13075-026-03830-w)
Supplement: Supplementary file 3 — Supplementary Material 3. Supplementary Table S1. Cardiovascular comorbidities and risk factors in patients with newly diagnosed IIM and patients with established IIM. [file 13075_2026_3830_MOESM3_ESM.docx]

**Supplementary Table S1.** Cardiovascular comorbidities and risk factors in patients with newly diagnosed IIM and patients with established IIM.

| **Parameter** | **Established IIM (*n* = 109)** | **Newly diagnosed IIM (*n* = 34)** |
| --- | --- | --- |
| BMI, kg/m^2^, median (IQR) | 26 (23-32) | 26 (22-30) |
| Ever smoker, n (%) | 42 (39) | 18 (53) |
| Comorbidities, n (%) |  |  |
| Hypertension | 34 (31) | 13 (38) |
| Diabetes mellitus | 24 (22) | 9 (26) |
| Dyslipidaemia | 43 (39.5) | 15 (44) |
| Obesity (BMI > 30 kg/m^2^) | 34 (31) | 7 (21) |
| Known cerebral vascular accident (stroke/TIA) | 4 (3.5) | 1 (3) |
| Known coronary heart disease | 11 (10) | 4 (12) |

BMI: body mass index; IIM: idiopathic inflammatory myopathy; IQR: interquartile range.
