# Supplementary material for: Elevated sST2 associates with cardiac involvement and declines after treatment in newly diagnosed patients with idiopathic inflammatory myopathies
Source: Arthritis Res Ther. 2026 May 19;28:109. doi: 10.1186/s13075-026-03830-w (PMC13185292; doi:10.1186/s13075-026-03830-w)
Supplement: Supplementary file 4 — Supplementary Material 4. Supplementary Table S2. Disease activity and treatment for 34 newly diagnosed IIM patients at baseline, 6 months,12 months and 24 months follow-up. [file 13075_2026_3830_MOESM4_ESM.docx]

**Supplementary Table S2**. Disease activity and treatment for 34 newly diagnosed IIM patients at baseline, 6 months,12 months and 24 months follow-up.

|  | Baseline | 6 months | 12 months | 24 months |
| --- | --- | --- | --- | --- |
| **Disease activity measures, median (IQR)** |  |  |  |  |
| Physician global disease activity VAS, 0-100 | 60 (47-60)^a**** b**** c****^ | 20 (0-22.5)^d** e**^ | 10 (0-10) | 0 (0-10) |
| Patient global disease activity VAS, 0-100 | 80 (67-82) ^a**** b**** c****^ | 60 (40-70)^d*** e***^ | 40 (25-60)^f*^ | 30 (10-50) |
| Extramuscular activity score VAS, 0-100 | 50 (20-60) ^a**** b**** c****^ | 15 (0-22.5)^d*** e***^ | 0 (0-10) | 0 (0-10) |
| HAQ-DI, 0-3 | 0.56 (0-1.28)^c*^ | 0.13 (0-0.41) | 0 (0-0.56) | 0 (0-038) |
| Manuel muscle testing, 0-80 | 75 (67-80)^a** b** c**^ | 80 (78-80) | 80 (80-80) | 80 (80-80) |
| CK, ukat/L | 16 (1.2-61.5)^a** b* c*^ | 1.5 (0.9-3.3) | 1.8 (1.1-4) | 1.5 (1-3.1) |
| **Medical therapy at assessment** |  |  |  |  |
| Prednisone dose (mg), median (IQR) | 55 (17-60) ^a**** b**** c****^ | 5 (4-10)^d* e**^ | 2.5 (0-5) | 0 (0-1.8) |
| Synthetic DMARD, n (%) | 30 (88) | 25 (83) | 26 (90) | 24 (83) |
| Rituximab, n (%) | 6 (18) | 3 (10) | 4 (14) | 3 (10) |
| Cyclophosphamide, n (%) | 5 (15) | 2 (7) | 0 | 0 |
| IVIG, n (%) | 3 (9) | 2 (7) | 0 | 0 |

IIM: Idiopathic inflammatory myopathies. ^a^ significant difference between IIM at baseline and 6 months. ^b^ significant difference between IIM at baseline and 12 months. ^c^ significant difference between IIM at baseline and 24 months.^d^  significant difference between IIM at 6months and 12 months. ^e^ significant difference between IIM at 6 months and 24 months. ^f^ significant difference between IIM at 12 months and 24 months. ^*^ *P*<0.05, ^**^*P*<0.01, ^***^*P*<0.001, ^****^*P*<0.0001.
